# Beyond Ischemia: The Rare Occurrence of Hemorrhagic Strokes in Pediatric Sickle Cell Anemia

**DOI:** 10.7759/cureus.70871

**Published:** 2024-10-05

**Authors:** Mohammed Mustafa, Muhanned Amawi, Mustafa M Altoonisi, Wessam Soliman, Mohamed Kamal, Ziad Asaad, Aseel Albalawi, Joud Alharbi, Akram M Awadalla, Mohamed A-Azim Ahmed, Ehab Hanafy

**Affiliations:** 1 Prince Sultan Oncology Center, King Salman Armed Forces Hospital, Tabuk, SAU; 2 Pediatrics, King Salman Armed Forces Hospital, Tabuk, SAU; 3 Pediatrics, king Salman Armed Forces Hospital, Tabuk, SAU; 4 Neurological Surgery, King Salman Armed Forces Hospital, Tabuk, SAU; 5 Radiology, King Salman Armed Forces Hospital, Tabuk, SAU

**Keywords:** epidural hemorrhage, mri, sickle cell anemia, stroke, subarachnoid hemorrhage, tcd

## Abstract

Sickle cell anemia (SCA) is a genetic disorder characterized by the production of abnormal hemoglobin S, leading to red blood cell sickling and subsequent vaso-occlusive events. Neurological complications, particularly strokes, significantly contribute to the morbidity and mortality associated with SCA. While ischemic strokes are more common, hemorrhagic strokes, though less frequent, present significant challenges, especially in the pediatric population. Understanding the complex interplay of genetic, environmental, and hematological factors is crucial for managing these cases.

We report two cases of pediatric patients with SCA who experienced rare hemorrhagic strokes. The first case involves a nine-year-old male presenting with a subarachnoid hemorrhage, revealing cortical ischemia and multiple cerebral artery strictures. Early supportive measures resulted in a good clinical improvement, after which the patient underwent bone marrow transplantation. The second case describes a seven-year-old male who developed an epidural hematoma during a vaso-occlusive crisis, necessitating emergency surgical intervention. After initial persistent neurological deficits, the patient began to show gradual improvement with ongoing management, reflecting the complexity and severity of such events.

Hemorrhagic strokes in pediatric SCA patients, though rare, represent significant clinical challenges due to their multifactorial etiology and complex management needs. These cases underscore the importance of a multidisciplinary approach and advanced diagnostic tools in managing hemorrhagic complications in SCA. Further research is essential to unravel the pathophysiological mechanisms and develop targeted prevention strategies to improve outcomes for this vulnerable population.

## Introduction

Sickle cell anemia (SCA) is a genetic hemoglobinopathy resulting from a mutation in the β-globin gene, leading to the production of abnormal hemoglobin S (HbS). This mutation causes red blood cells to assume a rigid, sickle-like shape, particularly under conditions of low oxygen tension. Cerebrovascular complications are a prominent concern in SCA, often resulting from both vasoconstriction and vaso-occlusion within cerebral vessels. Sickled red blood cells obstruct blood flow, leading to ischemic injury, while chronic inflammation and endothelial dysfunction further exacerbate vascular damage. Additionally, hemorrhagic events may occur due to weakened blood vessels, increasing the risk of strokes and other neurological deficits. These factors contribute significantly to the neurological morbidity associated with the disease [1,2].

Stroke is a devastating complication, occurring in approximately 11% of children with SCA by the age of 20 ​[1,2]. Strokes in SCA can be classified into ischemic and hemorrhagic types, with ischemic strokes being more prevalent in children, while hemorrhagic strokes are more common in adults. The incidence of hemorrhagic stroke in the pediatric SCA population is relatively low, estimated at around 3%, but the implications are serious, often resulting in long-term neurological deficits ​[2].

Hemorrhagic strokes in SCA patients can manifest as intracerebral hemorrhage (ICH), subarachnoid hemorrhage (SAH), epidural hematoma (EDH), and subdural hematoma (SDH). Each of these subtypes presents unique challenges in terms of pathophysiology, diagnosis, and management​ [2,3]. The risk factors for hemorrhagic stroke in this population include hypertension, previous ischemic stroke, moyamoya syndrome, cerebral aneurysms, and recent blood transfusion ​[1,3].

Despite advances in medical management and the introduction of screening protocols such as transcranial Doppler (TCD) ultrasound to detect cerebral vasculopathy, the prevention and treatment of hemorrhagic strokes in pediatric SCA patients remain complex​ [4]​. Recent studies highlight the role of magnetic resonance imaging (MRI) and magnetic resonance angiography (MRA) in identifying vascular abnormalities that predispose to hemorrhagic events​ [1,3].

In this report, we present two cases of pediatric patients with SCA who experienced hemorrhagic strokes, specifically a subarachnoid hemorrhage and an epidural hemorrhage. These cases underscore the need for heightened vigilance and a comprehensive approach to managing and preventing hemorrhagic complications in this vulnerable population.

## Case presentation

Case 1

A nine-year-old male with a known history of SCA was initially admitted due to a vaso-occlusive crisis (VOC). During his admission, he developed severe headaches, vomiting, and seizures. Additionally, he developed hypertension and was managed with hydralazine and amlodipine. He was also started on phenytoin and levetiracetam for seizure control. The patient had a history of acute chest syndrome (ACS), for which he had previously undergone exchange transfusion, as well as multiple prior admissions for VOC.

The patient was not compliant with hydroxyurea therapy. His developmental milestones were age-appropriate, his vaccinations were up to date and there was no history of previous surgeries.

**Figure 1:**
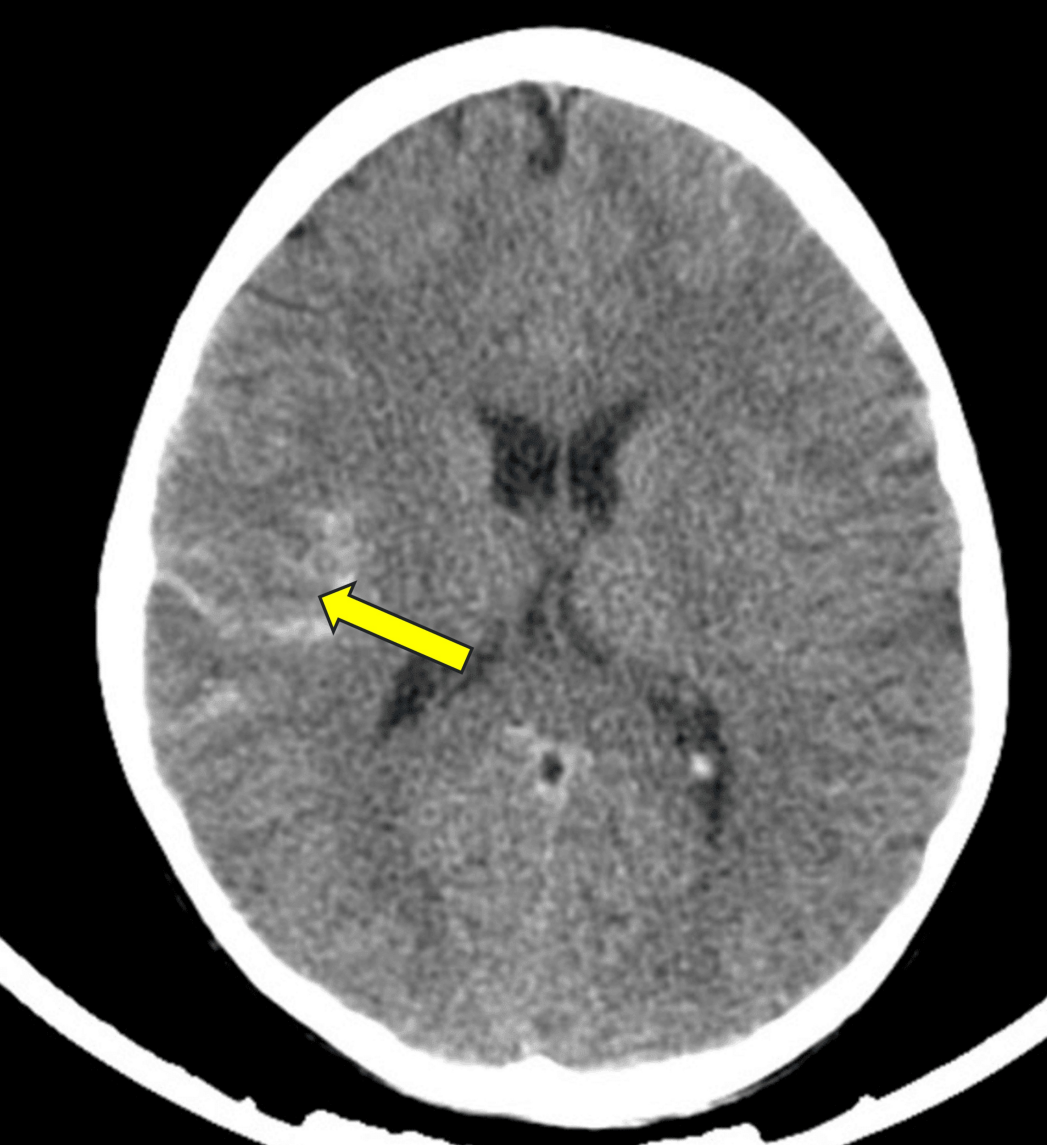
Non-contrast brain CT scan of case 1. The image demonstrates smearing of the right fronto-parietal and parietal cortical sulci by hyperdense material, indicative of subarachnoid hemorrhage.

Upon presentation, a computed tomography (CT) scan performed revealed evidence of SAH located at the upper right Sylvian fissure, the right high parietal sulci, the anterior interhemispheric fissure, and, to a lesser extent, the left frontal sulci and anterior left Sylvian fissure. The imaging also showed cortical edema and decreased density in the right high posterior parietal region, with effacement of the adjacent sulci suggestive of cortical ischemia. No midline shift, hydrocephalus, or space-occupying lesions (SOL) were identified. The adenoids were hypertrophied (Figure 1).

**Figure 2:**
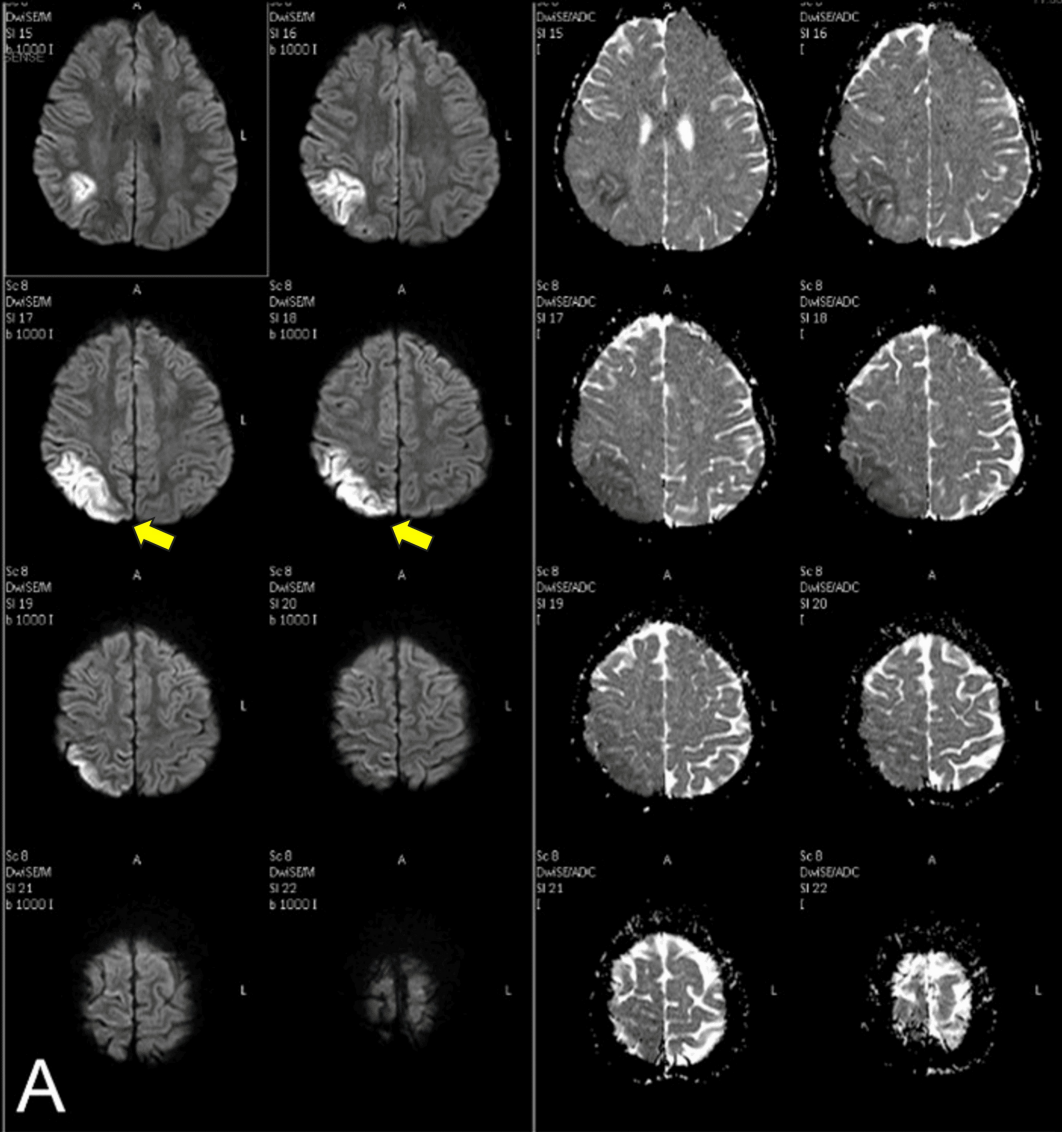
MRI examination of case 1. Diffusion-weighted imaging (DWI) and apparent diffusion coefficient (ADC) map show corresponding diffusion restriction in the right parietal cortical and subcortical region, consistent with an acute infarction.

Subsequent MRI and MRA scans of the brain (Figure 2) demonstrated cortical and subcortical edema with abnormal high T2 and FLAIR (fluid-attenuated inversion recovery) signal intensity and mild restricted diffusion in the right posterior parietal region, consistent with acute minor cortical ischemia. There was SAH with dark gradient signal intensity in the right high posterior parietal region, left frontal region, and right temporoparietal sulci, extending to the interhemispheric fissure. Multiple strictures were noted in the intercavernous and supraclinoid segments of the internal carotid arteries (ICA) bilaterally, with severe and diffuse narrowing of the right supraclinoid ICA and its branches (A1 & A2 of ACA {anterior cerebral artery} and M1 of MCA {middle cerebral artery}), which were less apparent on CT angiography. The left supraclinoid ICA showed diffuse narrowing, though to a lesser degree than the right, and was similarly less pronounced on CT angiography.

Additionally, the straight sinus was not visualized on magnetic resonance venography (MRV) but was noted in previous CTs with contrast. The anterior superior sagittal sinus (SSS) displayed areas not well recognized on MRV, appearing as diffuse narrowing. The posterior SSS showed similar characteristics, again more apparent on previous CTs. Diffuse narrowing of the lateral sinuses without filling defects was present, and there was notable collateralization with prominent superficial veins. No frank cerebral aneurysm or arteriovenous malformation (AVM) was identified.

The patient was admitted to the pediatric intensive care unit (PICU) for further management. His hemoglobin levels dropped during the hospital stay (Table 1), prompting a blood transfusion. Supportive care was provided, including hydration, pain management, antihypertensive and antiepileptic medications. Neurosurgical consultation was requested; however, surgical intervention was deemed unnecessary. Over the course of his admission, the patient showed gradual clinical improvement, and he was discharged in stable condition with no neurological deficits.

**Table 1:** Laboratory results of case 1 and case 2 at time of admission. WBC: white blood cells, RBC: red blood cells, HCT: hematocrit, MCV: mean corpuscular volume, PLT: platelet count, MPV: mean platelet volume, NEUT ABS: absolute neutrophils, LYMPH ABS: absolute lymphocytes, MONO ABS: absolute monocytes, EOS ABS: absolute eosinophils, BASO ABS: absolute basophils, ALP: alkaline phosphatase, AST: aspartate aminotransferase, ALT: alanine aminotransferase.

Variable	Unit	Reference range	Case 1	Case 2
Lactic acid dehydrogenase	U/L	141 - 237	1045	1043
WBC	10^3/µl	5.0 - 13.0	12.94	15.45
RBC	10^6/µl	4.0 - 5.0	2.43	1.94
Hemoglobin	g/dl	11.5 - 14	5.7	5.5
HCT	%	35.0 - 45.0	15.8	18
MCV	fl	77.0 - 88.0	65.0	92.8
PLT	10^3/µl	180 - 400	236	115
MPV	fl	7.0 - 13.0	8.5	10.3
NEUT ABS	10^3/µl	2.0 - 8.0	7.89	3.77
LYMPH ABS	10^3/µl	1.0 - 5.0	3.88	3.82
MONO ABS	10^3/µl	0.1 - 1.0	0.91	0.77
EOS ABS	10^3/µl	0.1 - 1.0	0.26	0.09
BASO ABS	10^3/µl	0.02 - 0.01	0.00	0
Reticulocyte %	%	0.5 - 2.5	27.3	25.9
Hemoglobin A quantitation	%	96 - 97.8	0.0	0
Hemoglobin A2 quantitation	%	2.2 - 3.7	3.3	1.9
Hemoglobin S quantitation	%	0.0 - 0.0	83.4	80.3
Hemoglobin F quantitation	%	<1.5	13.3	17.8
Sodium	mmol/L	136 - 145	136	142
Potassium	mmol/L	3.6 - 5.0	3.9	4.1
Chloride	mmol/L	98 - 107	105	105
Enzymatic bicarbonate	mmol/L	22 - 29	23	23
Urea nitrogen	mmol/L	1.6 - 4.6	2.22	3.1
Creatinine	µmol/L	20 - 70	28	29
Magnesium	mmol/L	0.61 - 0.90	0.81	0.7
Phosphate	mmol/L	1.00 - 1.90	1.45	1.44
Calcium	mmol/L	2.25 - 2.52	2.12	2.27
Total protein	g/L	63 - 81	76	88
Albumin	g/L	38 - 56	37	44
ALP	U/L	218 - 499	165	99
AST	U/L	15 - 34	119	62
ALT	U/L	12 - 49	39	24
Total bilirubin	µmol/L	5 - 21	30	44
Direct bilirubin	µmol/L	<5	5	10
Indirect bilirubin	µmol/L	2 - 9	25	34

A comprehensive neurological examination and systemic evaluation during admission revealed no remarkable findings. Given the evidence of cerebral ischemia observed on imaging, the patient was initiated on a regular transfusion program. Following successful bone marrow transplantation (BMT), the patient is now cured of SCA and currently maintains a good state of health.

Case 2

The second case involves a seven-year-old male with a known history of SCA. He has a significant history of frequent hemolytic crises and was previously under the care of another hospital before presenting to us. The child was admitted to our hospital due to a severe VOC, characterized by intense pain localized to the lower limbs and lumbar region, as well as abdominal pain and distension. Despite being on a regular treatment regimen of hydroxyurea and folic acid, his condition had acutely worsened.

On admission, the child was in distress. He exhibited stable vital signs and was afebrile, but he was notably jaundiced, with no respiratory or central nervous system symptoms initially. His pain was severe and inadequately controlled despite initial management with non-steroidal anti-inflammatory drugs, paracetamol, and opioids (morphine). Laboratory tests revealed leukocytosis, anemia with a hemoglobin level of 5.5 gm/dl, an elevated reticulocyte count, and a slightly low platelet count (Table 1).

On the following day, the patient's condition showed no significant improvement. He continued to suffer from severe pain and exhibited abdominal distension. The lack of immediate response to analgesics and supportive therapy prompted further investigation. His hemoglobin dropped further, and a blood transfusion was initiated.

Later, the patient developed a fever of 38.5°C, which was managed with antibiotics and antipyretics. After resuming and completing the blood transfusion, the patient initially showed slight improvement and normal vital signs. However, he subsequently experienced a sudden decline in neurological status, manifesting as decreased consciousness and lethargy.

**Figure 3:**
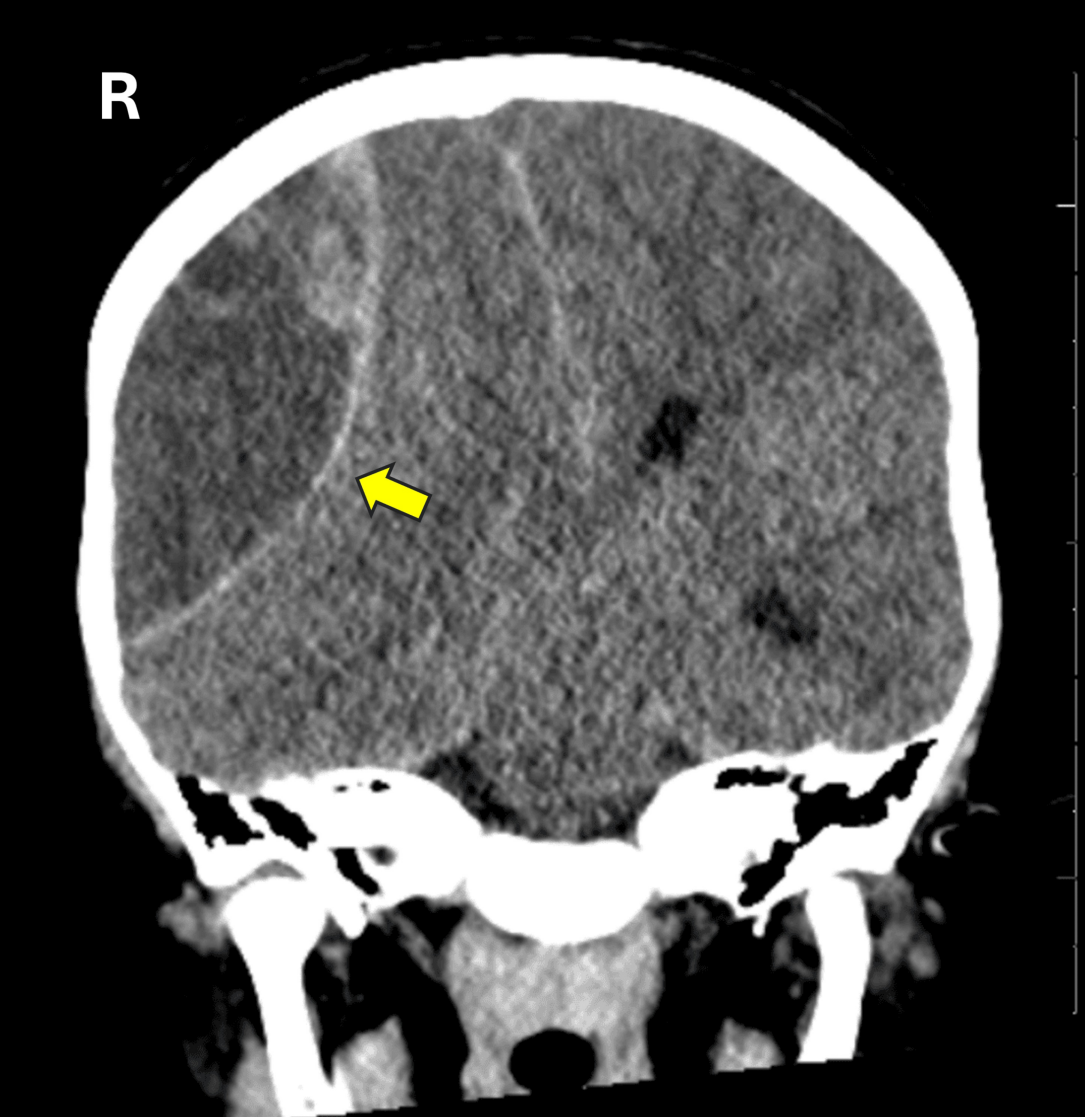
Non-contrast brain CT scan of case 2, coronal view. The images show a sizable right-sided extra-axial lentiform-shaped area of mixed density, with hyperdensity observed in the dependent portion, indicative of an extradural hematoma. There is a noticeable mass effect and midline shift.

A brain CT scan was urgently performed, revealing an epidural hematoma with significant midline shift, suggestive of acute intracranial hemorrhage (Figure 3). This alarming finding necessitated immediate intervention. The child was intubated to secure his airway, and a multidisciplinary team involving hematology, neurosurgery, and critical care specialists was engaged for further management.

In response to the clinical and imaging findings, the patient underwent emergency surgical intervention for evacuation of the hematoma. Despite the surgical intervention, the child remained intubated and in critical condition in the PICU. His postoperative course was complicated by persistent neurological deficits, necessitating ongoing intensive monitoring and supportive care.

**Figure 4:**
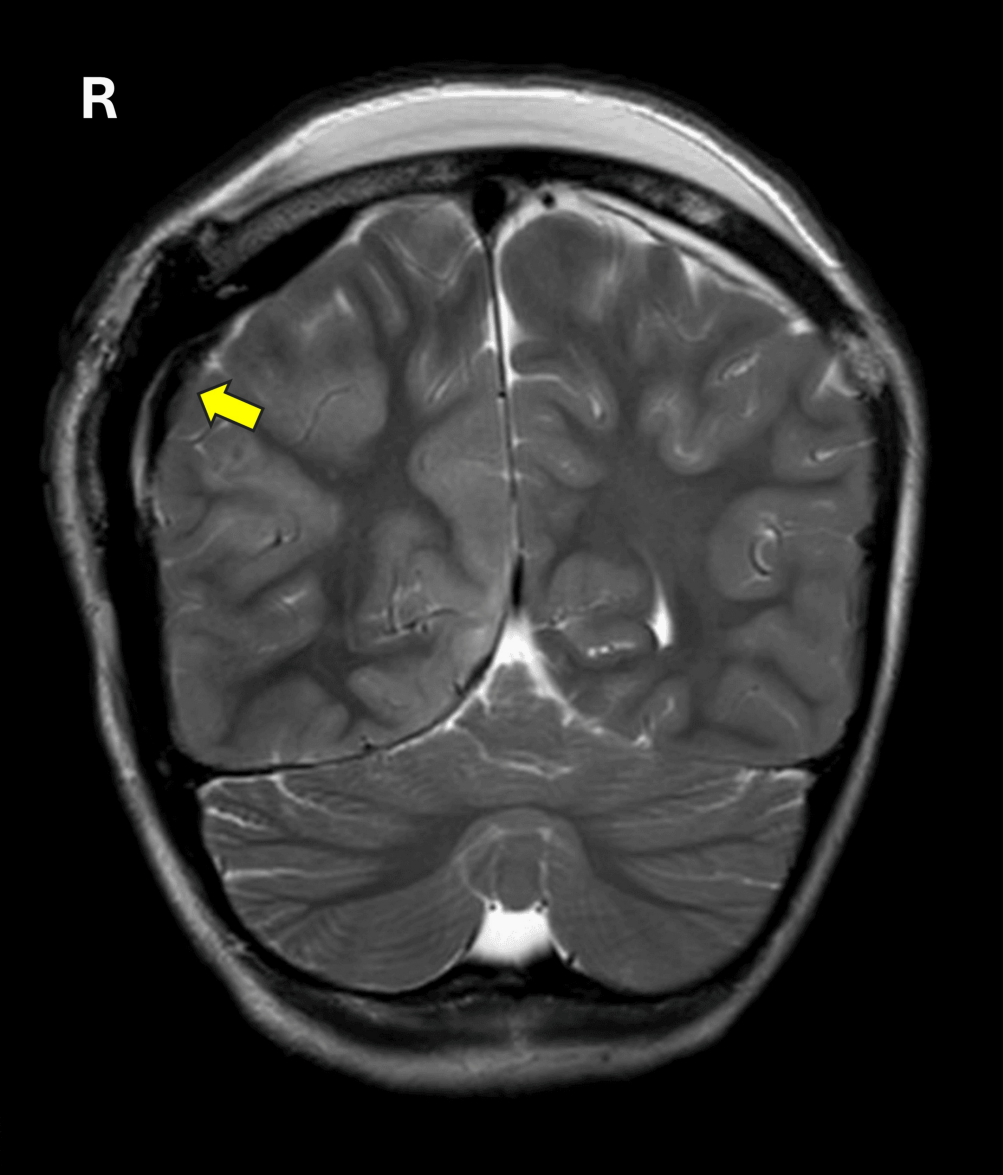
MRI examination of case 2 after evacuation of the extradural hematoma. Coronal T2 sequences demonstrate altered bone marrow signal intensity, involving the right and left sides of the skull. There is an associated subgaleal collection seen posteriorly, which is highly suggestive of acute soft head syndrome.

An MRI of the brain was conducted, showing multiple parenchymal areas of abnormal signal in the right occipital lobe, right frontal lobe, and several subcortical regions. The MRI findings were consistent with acute cerebral insult, likely secondary to vaso-occlusive phenomena associated with sickle cell disease (Figure 4). The MRI report further elucidated the severity of the condition, indicating no significant major cerebral arterial occlusion but suggesting acute soft head syndrome, a known complication in sickle cell disease. This condition contributes to neurological deterioration through vascular occlusion and ischemia, complicating an already severe clinical picture.

Throughout his PICU stay, laboratory studies continued to indicate a pattern consistent with disseminated intravascular coagulation (DIC), complicating his clinical picture with additional bleeding risks. His management required careful balancing of transfusion needs, and infection control measures, including broad-spectrum antibiotics to prevent sepsis.

During the PICU admission, with intensive supportive care, the child was extubated and began to show gradual improvement. A chronic transfusion program was planned due to the evidence of ischemia on MRI imaging.

This case illustrates the complex interplay of sickle cell disease and its severe complications, including VOC leading to life-threatening neurological events and systemic hematological disturbances.

## Discussion

SCA is a hereditary hemoglobinopathy characterized by the presence of sickle-shaped red blood cells. These cells are prone to causing vascular occlusions and subsequent ischemic damage due to their altered shape and function [5]. While ischemic strokes are more common in individuals with SCA, hemorrhagic strokes, though less frequent, represent a significant cause of morbidity and mortality, especially in the pediatric population [6]. Hemorrhagic strokes can manifest as SAH, ICH, or EDH, each presenting unique challenges in terms of diagnosis, management, and prevention [7].

The etiology of hemorrhagic stroke in patients with SCA is complex and multifaceted, involving a combination of genetic, hematological, and environmental factors. Each of these factors contributes uniquely to the risk profile of an individual with SCA, making the management and prevention of hemorrhagic strokes particularly challenging. Hemorrhagic strokes in SCA are often distinguished by characteristic findings such as chronic anemia, elevated white blood cell counts, and specific radiological patterns like cerebral vessel abnormalities or infarction, which help confirm the association with SCA rather than other causes.

Genetic predisposition plays a crucial role in determining the risk of hemorrhagic stroke in SCA patients. Several studies have identified specific genetic markers associated with an increased susceptibility to hemorrhagic events. For instance, the APOE-ε4 allele, which is more prevalent in individuals of African descent, has been linked to a higher risk of ICH among those with SCA [1,8,9]. This allele, known for its role in lipid metabolism, may contribute to cerebrovascular vulnerability in the context of sickle cell disease (SCD). Additionally, polymorphisms in genes such as ACE (angiotensin-converting enzyme), COL4A2 (type IV collagen alpha 2 chain), and MTHFR (methylenetetrahydrofolate reductase) have also been implicated in increasing the risk of hemorrhagic stroke [10]. The ACE gene, for example, is involved in blood pressure regulation, and certain variants may predispose individuals to hypertension, a known risk factor for stroke [1,2].

Environmental and physiological factors significantly contribute to the risk of hemorrhagic stroke in SCA patients. Hypertension, although traditionally not considered a major issue in SCD, has emerged as a significant concern and a potential risk factor for hemorrhagic stroke [1,9]. Chronic hemolytic anemia, a hallmark of SCA, leads to hyperdynamic circulation and increased cardiac output, which can predispose individuals to hypertension [1,2,9]. Over time, this increased vascular pressure can weaken blood vessel walls, making them more susceptible to rupture during hypertensive episodes. Additionally, lifestyle factors, including diet and physical activity, may also influence blood pressure levels and, consequently, stroke risk.

Hematological factors, including low steady-state hemoglobin concentration, high steady-state leukocyte count, and thrombocytopenia, further exacerbate the risk of bleeding in SCA patients. The chronic and recurrent VOC associated with SCA leads to endothelial damage and increased vessel fragility, setting the stage for potential hemorrhagic events [7]. The repeated sickling and unsickling of red blood cells contribute to microvascular damage, which can compromise the integrity of blood vessel walls. Furthermore, the increased use of hydroxyurea in SCA management, while beneficial in reducing the frequency of vaso-occlusive episodes, may have complex effects on platelet counts and function [8]. Hydroxyurea works by inducing fetal hemoglobin production, which reduces sickling; however, it can also affect bone marrow function and potentially alter the hemostatic balance, thereby influencing bleeding risk.

In our first case, the patient’s leukocytic count was not primarily elevated, however, he exhibited persistently low steady-state hemoglobin levels due to frequent hemolysis. In contrast, the second patient, despite not experiencing frequent crises, maintained hemoglobin levels ranging between 7.5 and 8.5 g/dL, with a steady-state leukocytic count within normal limits. Neither patient had a prior history of hypertension or other known risk factors for stroke, although the first patient developed hypertension during his admission. Additionally, the first patient was non-compliant with hydroxyurea treatment, whereas the second patient demonstrated adherence to the prescribed regimen.

Notably, the interplay of genetic, environmental, and hematological factors in SCA creates a complex landscape of risk for hemorrhagic stroke. Understanding these factors is essential for developing targeted interventions and preventive strategies aimed at reducing the burden of stroke in this vulnerable population. Ongoing research into the genetic underpinnings and pathophysiological mechanisms of stroke in SCA will be crucial in guiding future therapeutic approaches and improving outcomes for patients.

The pathophysiology of hemorrhagic stroke in SCA involves several interrelated mechanisms that collectively increase the risk of cerebrovascular events. Chronic hemolysis, a hallmark of SCA, results in the release of free hemoglobin into the bloodstream. This free hemoglobin binds to and scavenges nitric oxide (NO), a molecule essential for maintaining vascular tone and endothelial function [11]. The reduction in NO availability leads to endothelial dysfunction and increased vascular permeability, making blood vessels more susceptible to damage and rupture [12]. This process is a key contributor to the formation of aneurysms and other vascular malformations, such as moyamoya disease, which are frequently observed in SCA patients and serve as significant risk factors for hemorrhagic stroke [13,14].

In addition to the effects of hemolysis, the sickled red blood cells themselves play a direct role in promoting vascular injury. These abnormally shaped cells are rigid and prone to causing blockages in small blood vessels, leading to chronic vascular inflammation and oxidative stress [15]. The repeated episodes of vaso-occlusion and reperfusion injury associated with SCA exacerbate endothelial damage over time. This ongoing endothelial injury results in a cycle of inflammation and repair, which further weakens the vascular walls and increases the likelihood of hemorrhage [7,11]. The continuous oxidative stress generated by the presence of sickled cells contributes to the degradation of the vascular endothelium, compounding the risk of hemorrhagic events [14,15].

The presence of moyamoya arteriopathy, characterized by progressive stenosis of the intracranial vessels and the subsequent development of fragile collateral networks, adds another layer of complexity to the pathophysiology of hemorrhagic stroke in SCA [12,13]. Moyamoya disease is a condition where the cerebral blood vessels become increasingly narrowed, leading to the formation of a network of tiny, fragile vessels that attempt to compensate for reduced blood flow. These collateral vessels are prone to rupture, especially under the stress of increased blood pressure or other hemodynamic changes, thereby significantly raising the risk of both ischemic and hemorrhagic strokes in affected individuals [12,14].

Furthermore, the chronic inflammatory state induced by sickled red blood cells and the associated oxidative stress not only damages the endothelium but also disrupts the blood-brain barrier [11]. This disruption allows for the leakage of blood components into the brain parenchyma, exacerbating cerebral edema and increasing the potential for intracranial hemorrhage [14,15]. The repeated inflammatory insults and resultant vascular remodeling in SCA patients create a fragile cerebrovascular network that is highly susceptible to rupture under conditions of hemodynamic stress or hypertensive episodes [12].

Moreover, the hemodynamic changes associated with chronic anemia in SCA, including increased cardiac output and hyperdynamic circulation, place additional stress on the cerebral vasculature [11]. The compensatory mechanisms aimed at maintaining adequate tissue perfusion under anemic conditions lead to elevated blood flow velocities and shear stress on the vessel walls [15]. Over time, these hemodynamic forces contribute to the weakening of the vascular structures and predispose patients to hemorrhagic strokes [12,14].

Hemorrhagic stroke in pediatric SCA patients presents with a variety of clinical symptoms that are influenced by the location and severity of the hemorrhage. These symptoms are often sudden and severe, which necessitates prompt recognition and intervention to prevent further complications. The clinical manifestations of hemorrhagic stroke in SCA are diverse and can sometimes overlap with other complications of the disease, complicating the diagnostic process. Understanding these manifestations in detail is crucial for timely and effective management.

The most common initial symptom of a hemorrhagic stroke in pediatric SCA patients is a sudden, severe headache, often described by patients as the "worst headache of their life." This symptom is reported in approximately 60-80% of pediatric patients with intracranial hemorrhages [14,16]. The intensity of the headache may vary depending on the location of the hemorrhage. In cases of ICH, the headache is typically localized, while in SAH, it is often diffuse and associated with neck stiffness (nuchal rigidity) and photophobia due to meningeal irritation [14,16].

In our report, the first case presented with a severe headache, vomiting, seizures, and accompanied hypertension while the second case involved a sudden deterioration in consciousness with no preceding symptoms other than the pain associated with the vaso-occlusive crisis.

Vomiting and altered levels of consciousness are also common presentations. Approximately 40-60% of patients experience nausea and vomiting, which can occur suddenly and without warning [7]. Altered consciousness ranges from mild confusion to deep coma and is observed in 30-50% of pediatric cases with significant hemorrhagic events [14,16]. The severity of these symptoms often correlates with the extent and location of the bleed, with larger or more strategically placed hemorrhages (such as those affecting the brainstem) leading to more profound alterations in consciousness.

Focal neurological deficits, such as hemiparesis, aphasia, or sensory disturbances, are present in about 50-70% of pediatric patients with hemorrhagic strokes [16]. Hemiparesis is particularly common when the hemorrhage occurs in or near motor areas of the brain. These deficits can help localize the lesion but can also mimic symptoms of ischemic strokes or other complications like acute chest syndrome (ACS) or VOC [7,14]. This mimicry underscores the importance of differential diagnosis in the clinical setting.

The overlap in clinical presentation with other SCA-related complications, such as VOC, ACS, and severe infections, poses a significant challenge in early and accurate diagnosis. VOC, for example, can also present with severe pain and neurological symptoms due to infarcts or bone marrow expansion, potentially leading clinicians to misattribute the symptoms if not carefully evaluated [16,17].

Neuroimaging is essential in distinguishing hemorrhagic stroke from these other conditions. MRI and CT scans are the primary tools used for diagnosis. MRI is particularly valuable due to its sensitivity in detecting subtle changes in brain tissue, including those caused by small hemorrhages and associated edema [7,18]. It can identify characteristic features such as the presence of hemosiderin deposits from previous bleeds or evidence of moyamoya disease, which is seen in approximately 6-8% of pediatric SCA patients [16].

CT scans, while less sensitive than MRI in detecting certain types of hemorrhages, are more readily available and can rapidly identify acute bleeding, making them invaluable in emergency settings [7]. In a study involving pediatric SCA patients, CT scans were used to confirm the diagnosis of hemorrhagic stroke in over 90% of acute cases, highlighting their crucial role in initial assessment [17].

The MRI in our study of the second case revealed an additional finding of soft head syndrome with widespread abnormal signal intensity at the left parietal bone. While epidural hemorrhage is typically associated with traumatic events, which is not the case for our patient, the presence of soft head syndrome may serve as a precipitating factor. Soft head syndrome is characterized by the softening of the scalp due to vaso-occlusive crises and bone marrow expansion, which can lead to cortical disruption, osteonecrosis, and reactive subgaleal collections. This condition, combined with the vascular fragility inherent in sickle cell anemia, may increase the likelihood of spontaneous epidural hematoma formation, even without a clear history of trauma.

The management of hemorrhagic stroke in patients with SCA necessitates a comprehensive, multidisciplinary approach tailored to the specific needs of each patient. This complex condition requires the coordinated efforts of various healthcare professionals, including hematologists, neurologists, neurosurgeons, intensivists, and rehabilitation specialists, to optimize outcomes and minimize complications [19,20]. Immediate management goals focus on stabilizing the patient, controlling intracranial pressure, preventing rebleeding, and addressing the underlying pathophysiological processes associated with SCA.

Stabilization of the patient is the first and foremost priority in the acute setting. This involves ensuring airway patency, adequate breathing, and circulation. Intravenous access should be secured for the administration of medications and fluids, and continuous monitoring of vital signs is essential to detect any signs of deterioration [7,14]. In cases of severe hemorrhagic stroke, patients may require intubation and mechanical ventilation to protect the airway and support respiratory function [21].

Controlling intracranial pressure (ICP) is a critical aspect of managing hemorrhagic stroke. Elevated ICP can lead to brain herniation and further neurological damage, so timely intervention is crucial [7,14]. Medical management typically includes the use of osmotic agents such as mannitol or hypertonic saline to reduce cerebral edema and lower ICP [21,22]. These agents work by drawing fluid out of the brain tissue, thereby reducing swelling and relieving pressure on the brain. In addition to osmotic therapy, maintaining the head of the bed at a 30-degree angle can also aid in reducing ICP by promoting venous drainage [19].

In our first case, anticonvulsants were necessary to prevent and manage seizures, a common complication of hemorrhagic stroke caused by the irritation of the cerebral cortex by blood products [22]. Antiepileptic drugs such as levetiracetam or phenytoin can be used to reduce the risk of seizures and prevent further neurological injury [21].

Surgical intervention is often required in cases where there is a significant mass effect, midline shift, or continued bleeding despite medical management [19]. Procedures such as craniotomy or hematoma evacuation can be lifesaving by alleviating pressure on the brain and removing the source of bleeding [7]. The decision to proceed with surgery depends on the location and size of the hemorrhage, as well as the patient's overall clinical status [21]. In some cases, endovascular techniques such as coiling, or embolization may be used to manage aneurysms or vascular malformations that are contributing to the hemorrhage [7,14].

In our study, the first case was managed conservatively using supportive measures, eliminating the need for surgical intervention. In contrast, the second case required surgical evacuation of the hematoma in addition to supportive care, which included respiratory support and inotropes. This underscores the importance of tailoring the treatment plan to each patient's unique clinical circumstances, highlighting the need for an individualized approach to management.

In the context of SCA, blood transfusions play a pivotal role in managing hemorrhagic stroke. Transfusions help to reduce the proportion of sickle hemoglobin (HbS) in the circulation, thereby decreasing the risk of further sickling and improving oxygen delivery to tissues [14,21]. Exchange transfusions are particularly effective in rapidly lowering HbS levels and can be beneficial in severe cases. However, they must be used cautiously and tailored to individual patients, as they can cause additional harm if not appropriately indicated or performed correctly. Rapid changes in blood volume and viscosity during the procedure may affect intracranial pressure, potentially worsening neurological outcomes. Additionally, alterations in electrolytes and fluid balance can increase the risk of seizures in patients already vulnerable due to a hemorrhagic event [7,22].

Hydroxyurea, a medication traditionally used to prevent VOC and ischemic strokes, may also have a role in reducing the frequency of hemorrhagic episodes in SCA patients [19,21]. Hydroxyurea works by increasing fetal hemoglobin (HbF) production, which reduces the overall proportion of sickle hemoglobin and ameliorates the sickling process [14]. Its potential benefits in hemorrhagic stroke management are still being explored, but it may contribute to improved vascular stability and reduced inflammation [23].

Emerging therapies offer promising avenues for modifying the disease course and reducing the risk of both ischemic and hemorrhagic strokes in SCA. Gene therapy, which aims to correct the underlying genetic defect in sickle cell anemia, is currently under investigation and has shown potential in preclinical and early clinical trials [19,22]. By introducing healthy copies of the beta-globin gene or using genome editing techniques to reactivate fetal hemoglobin production, gene therapy has the potential to cure SCA and eliminate its complications [20].

Hematopoietic stem cell transplantation (HSCT) is another potential curative approach for SCA that has demonstrated efficacy in reducing the risk of stroke and other complications [7]. HSCT involves replacing the patient's diseased bone marrow with healthy donor stem cells capable of producing normal red blood cells [24]. While HSCT has shown promise, it is associated with significant risks, including graft-versus-host disease and long-term immunosuppression, and is therefore typically reserved for severe cases or patients with matched sibling donors [22,24]. Ongoing research is focused on improving the safety and accessibility of HSCT, including the use of alternative donor sources and reduced-intensity conditioning regimens [19].

However, these emerging treatments require further research to establish their efficacy and safety in the pediatric population. Clinical trials are ongoing, and it is hoped that these innovative therapies will eventually become standard care for managing SCA and preventing its complications [7,19]. Until these treatments become widely available, the management of hemorrhagic stroke in SCA will continue to rely on established medical and surgical interventions, tailored to the individual needs of each patient [21,22].

The prognosis of hemorrhagic stroke in pediatric sickle cell anemia (SCA) patients is variable and depends on several factors, including the severity of the hemorrhage, the presence of underlying vascular abnormalities, and the timeliness of intervention [7,16]. Studies have shown that while the overall survival rates have improved with advances in medical care, long-term neurological outcomes remain a concern [24]. Complications such as cognitive impairment, motor deficits, and recurrent strokes are not uncommon and necessitate ongoing rehabilitation and support [17,20].

The severity of the hemorrhage plays a significant role, as severe hemorrhages leading to extensive brain damage are associated with a poorer prognosis compared to milder cases [17,25]. Additionally, the presence of vascular abnormalities, such as moyamoya syndrome or aneurysms, can complicate the course of the disease and worsen outcomes. Children with these conditions are at higher risk for recurrent strokes, requiring vigilant monitoring and treatment [14,16]. The timeliness of intervention is also crucial, as early intervention and management can significantly improve outcomes by mitigating damage and enhancing recovery prospects [20,21].

In terms of long-term outcomes, cognitive impairment is a common challenge faced by many children who experience hemorrhagic stroke, impacting their academic performance and overall quality of life [23,24]. Motor deficits, such as hemiparesis or other impairments, often require long-term rehabilitation to maximize recovery and function [7]. Furthermore, there is a significant risk of recurrent strokes in this population, necessitating ongoing preventative strategies such as regular blood transfusions or hydroxyurea therapy to reduce the risk and improve patient outcomes [14,17].

## Conclusions

Hemorrhagic strokes in pediatric patients with sickle cell anemia, although rare, represent complex and life-threatening events. These cases demonstrate the critical role of early recognition, intensive supportive care, and multidisciplinary management in improving outcomes. While bone marrow transplantation offers a curative option, regular transfusion programs remain essential in mitigating ischemic risks. Further research is crucial to better understand the underlying pathophysiology and to develop effective preventive strategies for managing these challenging complications in sickle cell disease.
